# Characterization of E3 ligases involved in lysosomal sorting of the HIV-1 restriction factor BST2

**DOI:** 10.1242/jcs.195412

**Published:** 2017-05-01

**Authors:** Nicolas Roy, Grégory Pacini, Clarisse Berlioz-Torrent, Katy Janvier

**Affiliations:** 1 Inserm, U1016, Institut Cochin, Paris, France; 2 CNRS, UMR8104, Paris, France; 3 Université Paris Descartes, Sorbonne Paris Cité, Paris, France

**Keywords:** BST2, Tetherin, Ubiquitylation, NEDD4, MARCH8, β-TrCP, Vpu

## Abstract

The cellular protein BST2 (also known as tetherin) acts as a major intrinsic antiviral protein that prevents the release of enveloped viruses by trapping nascent viral particles at the surface of infected cells. Viruses have evolved specific strategies to displace BST2 from viral budding sites in order to promote virus egress. In HIV-1, the accessory protein Vpu counters BST2 antiviral activity and promotes sorting of BST2 for lysosomal degradation. Vpu increases polyubiquitylation of BST2, a post-translation modification required for Vpu-induced BST2 downregulation, through recruitment of the E3 ligase complex SCF adaptors β-TrCP1 and β-TrCP2 (two isoforms encoded by *BTRC* and *FBXW11*, respectively). Herein, we further investigate the role of the ubiquitylation machinery in the lysosomal sorting of BST2. Using a small siRNA screen, we highlighted two additional regulators of BST2 constitutive ubiquitylation and sorting to the lysosomes: the E3 ubiquitin ligases NEDD4 and MARCH8. Interestingly, Vpu does not hijack the cellular machinery that is constitutively involved in BST2 ubiquitylation to sort BST2 for degradation in the lysosomes but instead promotes the recognition of BST2 by β-TrCP proteins. Altogether, our results provide further understanding of the mechanisms underlying BST2 turnover in cells.

## Abstract (precis)

**Highlighted Article:** We identify two E3 ubiquitin ligases, NEDD4 and MARCH8, as regulators of BST2 (tetherin) – a protein that restricts viral release; we thus provide further understanding of the mechanisms underlying BST2 turnover in cells.

## INTRODUCTION

The interferon-inducible cellular protein ‘bone marrow stromal antigen 2’ (BST2; also known as tetherin, CD317 or HM1.24) is an intrinsic antiviral factor that restricts the release of many enveloped viruses, such as retroviruses, herpes viruses, filoviruses, rhabdoviruses, paramyxoviruses and arenaviruses, making it an essential component of the innate immune defence against viral dissemination (Neil, 2013; Tokarev et al., 2009).

Although upregulated by type-I interferon and pro-inflammatory stimuli, BST2 is constitutively expressed in several cell types as a 30- to 36-kDa type II integral membrane glycoprotein, present both at the plasma membrane and in intracellular compartments such as the trans-Golgi network (TGN) and early/recycling endosomes. BST2 contains a short N-terminal cytoplasmic tail linked to a transmembrane domain and a large extracellular coiled-coil domain anchored to the membrane through a C-terminal glycosyl-phosphatidylinositol (GPI) moiety (Evans et al., 2010; Kupzig et al., 2003; Masuyama et al., 2009). This atypical topology is responsible for its restrictive function towards viral release; BST2 assembles as parallel disulfide-bonded homo-dimers and acts as a bridge between virions and cellular membranes via its GPI anchors and its transmembrane domain, respectively, leading to the retention of the nascent viral particles at the surface of infected cells (Iwabu et al., 2009; Neil et al., 2008; Perez-Caballero et al., 2009; Van Damme et al., 2008; Venkatesh and Bieniasz, 2013).

In order to efficiently egress from cells, viruses have evolved strategies to displace BST2 from viral assembly sites in such a way as to counteract its antiviral activity (Evans et al., 2010; Neil, 2013; Roy et al., 2014). Several virus-encoded proteins devoted to this function have been characterized, notably Vpu of human immunodeficiency virus type 1 (HIV-1) (Neil et al., 2008; Van Damme et al., 2008), Nef of most strains of simian immunodeficiency virus (SIV) and of HIV-1 Group O (Jia et al., 2009; Kluge et al., 2014; Sauter et al., 2009; Zhang et al., 2009), envelope glycoproteins (Env) of HIV-2 and SIVtan, (Gupta et al., 2009; Le Tortorec and Neil, 2009) and K5 of Kaposi's sarcoma-associated herpes virus (KSHV) (Mansouri et al., 2009).

Antagonism of BST2 by lentiviral Vpu, Env and Nef relies on an alteration of BST2 intracellular trafficking though a complex interplay with host components that regulate protein vesicular transport, leading ultimately in most cell types to an overall decrease of BST2 at the surface of infected cells (Goffinet et al., 2009; Iwabu et al., 2009; Le Tortorec and Neil, 2009; Van Damme et al., 2008). HIV-1 Vpu has been notably shown to prevent the recycling of BST2 that has been internalized from the cell surface, as well as access to the plasma membrane (PM) of *de novo* synthetized BST2, thereby decreasing the resupply of BST2 to the PM (Dubé et al., 2011; Lau et al., 2011; Schmidt et al., 2011).

Vpu-induced downregulation of cell surface BST2 is also associated with enhanced targeting of the restriction factor for lysosomal degradation (Douglas et al., 2009; Iwabu et al., 2009; Mitchell et al., 2009). Both we and others have shown that Vpu promotes ESCRT-mediated sorting of BST2 for lysosomal degradation (Agromayor et al., 2012; Janvier et al., 2011). The ESCRT machinery regulates sorting of ubiquitylated membrane proteins to the multivesicular bodies (MVBs) for their subsequent degradation in lysosomes (Raiborg and Stenmark, 2009). Interestingly, BST2 undergoes ubiquitylation (Gustin et al., 2012; Pardieu et al., 2010; Tokarev et al., 2010) through a not fully characterized process, and Vpu has been reported to induce increased polyubiquitylation of BST2 on serine (S3, S5) and threonine (T4) residues located in its cytoplasmic tail (Tokarev et al., 2010). However, numerous questions remain regarding the significance of BST2 ubiquitylation on its constitutive trafficking and sorting for degradation, and there are contradictory results concerning the contribution of polyubiquitylation of BST2 S3-T4-S5 residues on Vpu-induced degradation of BST2 and viral egress (Cocka and Bates, 2012; Gustin et al., 2012; Tokarev et al., 2010). Polyubiquitylation of BST2 by Vpu is mediated by the recruitment of the substrate-recognition subunits of the Skp1–Cullin1–F-Box (SCF) E3 ligase, the β-TrCP proteins (encoded by *BTRC* and *FBXW11*; also known as β-TrCP1 and β-TrCP2, respectively; hereafter collectively referred to as β-TrCP), via a ^51^DSGxxS^56^ consensus motif (where x indicates any amino acid) located in the cytoplasmic tail of Vpu (Gustin et al., 2012; Tokarev et al., 2010). β-TrCP is required for Vpu-mediated BST2 downregulation at the cell surface and its targeting for endo-lysosomal degradation (Douglas et al., 2009; Iwabu et al., 2009; Mangeat et al., 2009; Mitchell et al., 2009). However, the requirement of β-TrCP in Vpu antagonism of BST2 antiviral activity remains controversial (Iwabu et al., 2009; Kueck et al., 2015; Mangeat et al., 2009; Mitchell et al., 2009; Tervo et al., 2011). Furthermore, the importance of β-TrCP in the constitutive ubiquitylation and turnover of BST2 has not been addressed.

In this study, we investigated the mechanisms underlying BST2 ubiquitylation and its role in the regulation of BST2 trafficking and turnover. To this end, we performed a small siRNA screen to identify the E3 ligases involved, and then unraveled the role of two specific E3 ligases: the HECT E3 ligase NEDD4 and the membrane-associated RING-CH 8 (MARCH8) E3 ligase, in the regulation of BST2 constitutive ubiquitylation and turnover. Depletion of NEDD4 and MARCH8 in cells resulted in altered ubiquitylation of BST2 associated with a delayed turnover of the restriction factor. Conversely, their overexpression caused an overall increase of BST2 ubiquitylation. However, NEDD4 and MARCH8 do not contribute to Vpu-induced downregulation of BST2. In accordance with previous studies, we show that Vpu modulates BST2 expression through recruitment of β-TrCP, which does not contribute to regulation of constitutive ubiquitylation of BST2. This suggests that Vpu bypasses, to some extent, the machinery involved in the constitutive turnover of BST2 to direct the restriction factor for degradation.

## RESULTS

### MARCH8, NEDD4 or β-TrCP depletion leads to increased levels of BST2

To characterize the E3 ligase(s) involved in the constitutive ubiquitylation of BST2 and its sorting for lysosomal degradation, we performed a small siRNA screen targeting a subset of well-characterized E3 ligases involved in the regulation of protein trafficking in the endo-lysosomal pathway: the HECT-E3 ligases NEDD4, NEDD4-L, ITCH, WWP1 (Boase and Kumar, 2015; Ingham et al., 2004); the RING domain-containing E3 ligases c-Cbl and the transmembrane protein MARCH8 (Goh and Sorkin, 2013; Ohmura-Hoshino et al., 2006a). We also included a well-characterized siRNA that targets both β-TrCP1 and β-TrCP2 (referred to herein as β-TrCP), which function as adaptors of the SCF E3 ligase and contribute to Vpu-induced sorting of BST2 for lysosomal degradation (Douglas et al., 2009; Iwabu et al., 2009; Mitchell et al., 2009). The previously described control siRNA (siCD) (Janvier et al., 2011) and an irrelevant siRNA targeting the E3 ligase RNF138 that is not expressed in HeLa cells (data not shown) were used as negative controls.

**Fig. 1.**
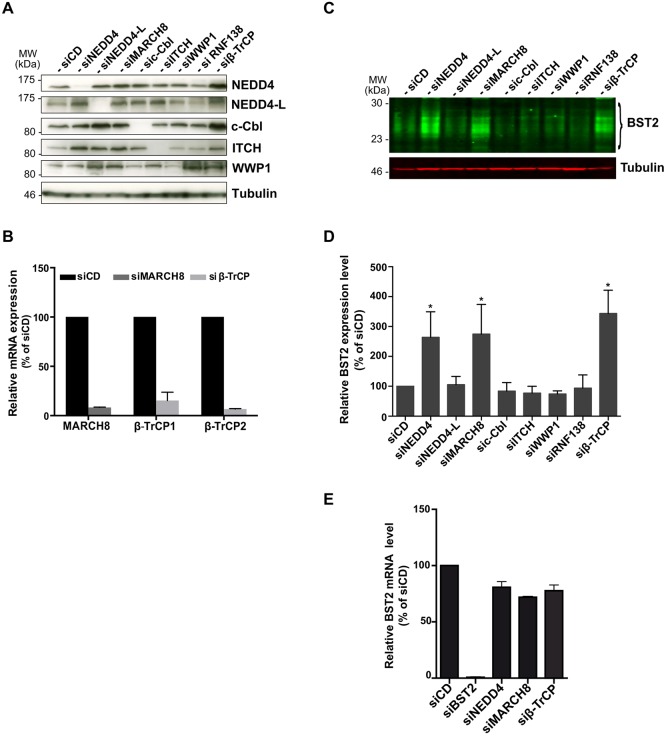
**Silencing of NEDD4, MARCH8 or β-TrCP induces enhanced levels of BST2.** (A,B) Analysis of E3-ubiquitin ligase depletion. HeLa cells transfected with the indicated siRNA or siRNA control (siCD) were lysed, and protein depletion was confirmed (A) by western blot analysis or (B) by RT-qPCR. (C,D) Impact of E3 ligase depletion on the cellular level of BST2. 20 µg of protein for each sample was loaded, and BST2 levels were assessed by quantitative western blotting. Tubulin is the loading control (C). BST2 relative levels were measured using ImageJ software and normalized to tubulin levels (D). Values were normalized to those obtained for the control cells set as 100%. Data are represented as mean±s.d. from three independent experiments (*n*=3). *P*-values were calculated using Student's *t-*test (compared with control cells). Significant results (*P<0.05) are indicated. (E) Impact of NEDD4, MARCH8 and β-TrCP depletion on BST2 transcription level. BST2 mRNA levels were quantified in siRNA-treated cells by performing RT-qPCR using specific primers. Values calculated were normalized to those obtained for control cells set as 100%. Bars represent the mean±s.d. (*n*=3).

We first evaluated the outcome of depletion of the abovementioned ligases on the expression level of BST2. HeLa cells were transfected with the selected siRNA, or siCD, and left for 4 days to allow significant depletion of the targeted proteins. Depletion was confirmed by western blotting (Fig. 1A) or by real-time quantitative PCR (RT-qPCR) (Fig. 1B). Steady-state BST2 levels in siRNA-treated cells were then assessed by quantitative western blotting and normalized to the amount of tubulin, used as a loading control (Fig. 1C,D). No major alteration in the amount of BST2 was observed upon knockdown of the E3 ligases NEDD4-L, ITCH, WWP1 or c-Cbl compared to control cells (Fig. 1). On the contrary, depletion of NEDD4, MARCH8 and β-TrCP resulted in a marked increase in the amount of BST2 detected in cell lysates, suggesting a role for these enzymes in the regulation of BST2 levels. Quantification of *BST2* transcripts in cells depleted of NEDD4, MARCH8 or β-TrCP was further assessed by performing RT-qPCR (Fig. 1E) and showed no significant difference compared to control cells. This suggests that the augmentation of BST2 was not due to increased *BST2* transcription but was most likely the result of post-transcriptional stabilization of the BST2 protein.

### MARCH8 and NEDD4 regulate ubiquitylation of BST2

**Fig. 2.**
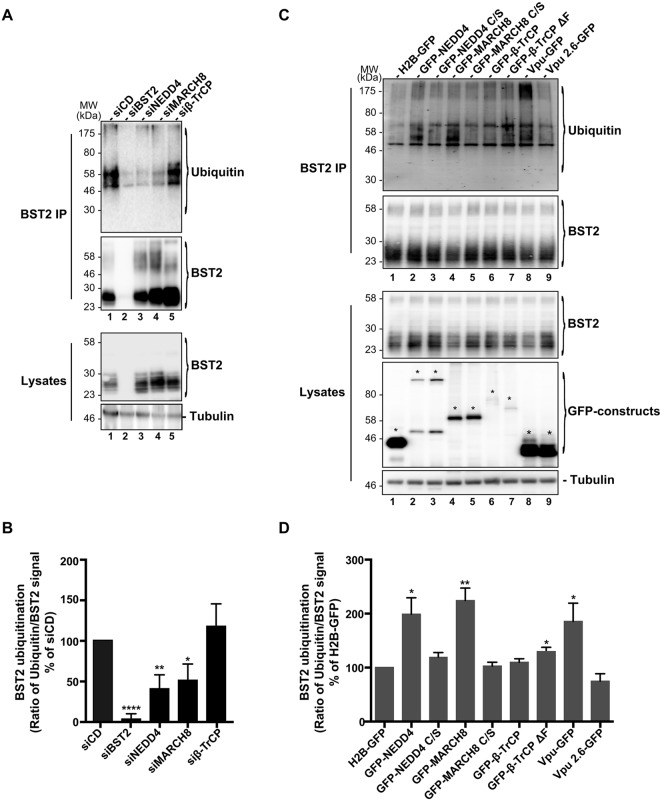
**Contribution of NEDD4, MARCH8 and β-TrCP to BST2 ubiquitylation.** (A,B) siRNA-transfected HeLa cells or (C,D) HeLa cells transfected with plasmid encoding wild-type (lanes 2, 4, 6) or catalytically inactive mutants (lanes 3, 5, 7) of NEDD4, MARCH8 or β-TrCP fused to GFP were lysed in stringent buffer, and BST2 was immunoprecipitated (IP) with an antibody against BST2. Ubiquitylation of BST2 was analyzed by western blot using an antibody against ubiquitin. In panels C,D, plasmids encoding GFP-tagged histone H2B (H2B–GFP), or WT Vpu (Vpu–GFP) or Vpu mutated on residues S52 and S56 (Vpu2.6–GFP) were used as controls. Asterisks in panel C indicate the bands corresponding to GFP fusion proteins. (B,D) Signals obtained for ubiquitin staining were normalized to those obtained for BST2. Values obtained for each condition were normalized to those obtained for control cells (siCD and H2B–GFP, respectively) set as 100%. Bars represent the mean±s.e.m. (*n*=4), ****P<0.0001, **P<0.01, *P<0.05.

We next tested whether NEDD4, MARCH8 or β-TrCP is involved in BST2 ubiquitylation (Fig. 2). To this end, siRNA-treated HeLa cells were lysed under stringent conditions (cf. Materials and Methods) and subjected to immunoprecipitation of BST2. Western blot analysis of the immunoprecipitated proteins using an antibody against ubiquitin revealed a few stacked bands between 35 kDa and 60 kDa that probably correspond to mono- and multi-ubiquitylated forms of BST2 (these bands will be referred to as such hereafter) (Fig. 2A, lane 1). In addition, smeared higher molecular bands could also be detected at a lower intensity and might represent previously described polyubiquitylated BST2 (Gustin et al., 2012; Tokarev et al., 2010). Analysis of BST2 ubiquitylation in cells that had been depleted for each of the E3 ligases showed a significant decrease in the amount of mono- and multi-ubiquitylated forms of BST2 upon depletion of NEDD4 and MARCH8 compared to those in control cells (siCD) (Fig. 2A,B, lanes 3,4). These results suggest that both NEDD4 and MARCH8 contribute to BST2 ubiquitylation. Surprisingly, β-TrCP knockdown did not significantly alter BST2 mono- and multi-ubiquitylation, suggesting that β-TrCP expression is not strictly required for constitutive ubiquitylation of BST2 (Fig. 2A,B, lane 5).

To confirm these results, we analyzed the effects of overexpressing wild-type (WT) and catalytically impaired mutant forms of the E3 ligases on the profile of BST2 ubiquitylation (Fig. 2C,D). Cysteine at position 867 in NEDD4 and positions 80, 83, 123 and 126 in the RING domain of MARCH8, which are required for catalytic activity, were mutated to serine (referred to hereafter as NEDD4 C/S and MARCH8 C/S), as previously described (Anan et al., 1998; Ohmura-Hoshino et al., 2006b). The well-characterized mutant of β-TrCP devoid of its F-box domain (β-TrCPΔF) was used as catalytically dead mutant for this enzyme (Winston et al., 1999). The ubiquitylation profile of BST2 was then assessed in HeLa cells that had been transfected with GFP-tagged WT, or mutated NEDD4, MARCH8 or β-TrCP. Overexpression of NEDD4 induced an increase in the level of mono- and multi-ubiquitylated BST2 compared to in control cells expressing histone2B–GFP (H2B–GFP) (Fig. 2C,D, lane 2 vs 1). This effect was abrogated upon expression of the catalytically impaired mutant NEDD4 C/S, confirming the relevance of NEDD4 for BST2 ubiquitylation (lane 3). Similarly, overexpression of MARCH8 increased BST2 mono- and multi-ubiquitylation (lane 4), whereas mutation of the catalytic cysteine residues of MARCH8 (MARCH8 C/S) abolished this effect (lane 5), also supporting a role of this E3 ligase in BST2 ubiquitylation. In line with the siRNA-based experiments (Fig. 2A,B), overexpression of WT β-TrCP or the negative mutant β-TrCPΔF did not potently alter the ubiquitylation profile of BST2 compared to that in control cells (lanes 6, 7 vs 1). These data are consistent with the notion that β-TrCP is not essential for constitutive ubiquitylation of BST2 and that the increased amount of BST2 observed in β-TrCP knockdown cells does not result from impaired BST2 ubiquitylation.

### Analysis of the NEDD4 and MARCH8 interaction with BST2

**Fig. 3.**
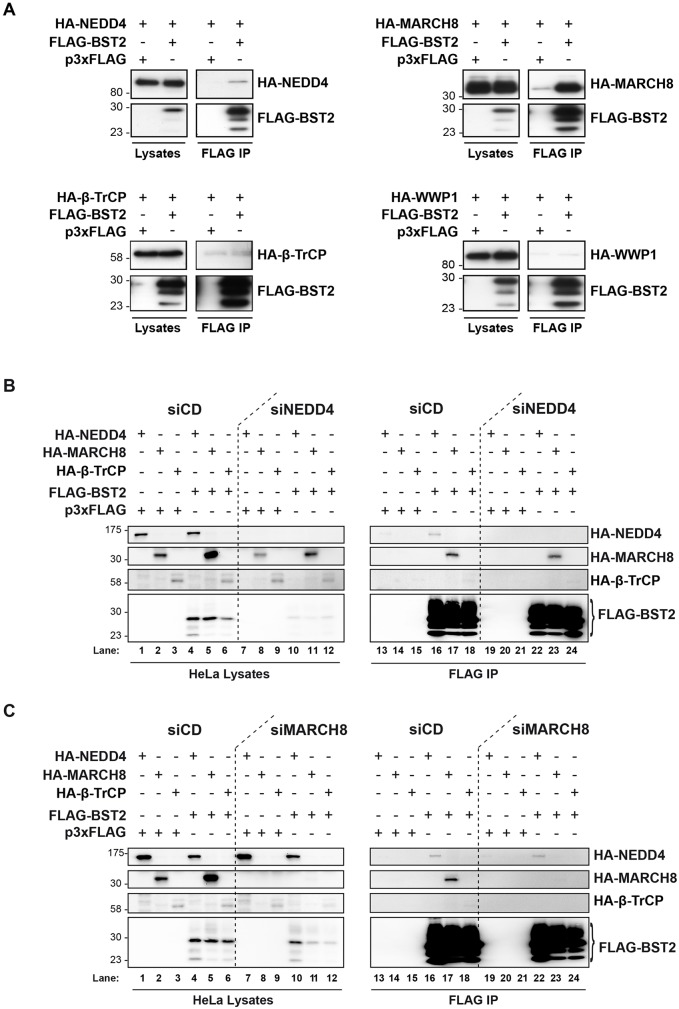
**Analysis of BST2 interaction with NEDD4, MARCH8 and β-TrCP.** (A) HEK293T cells were transfected with plasmid encoding FLAG–BST2 or p3xFLAG vector (used as negative control) along with expression vectors for HA–NEDD4, HA–MARCH8 or HA–β-TrCP, or HA–WWP1 used as negative control. Binding of HA-tagged E3 ubiquitin ligases and FLAG–BST2 was assessed by immunoprecipitating (IP) FLAG–BST2 followed by western blot analyses. Left panels represent the input (lysates) and right panels represent bound proteins (FLAG-IP). Data are representative of four independent experiments. (B,C) NEDD4 and MARCH8 interact with BST2 independently of each other. HeLa cells transfected with the indicated siRNA were transfected with both plasmids encoding FLAG–BST2 and HA-tagged E3 ligases, followed by FLAG–BST2 pull-down. Binding of HA-tagged E3 ubiquitin ligases and FLAG–BST2 was analyzed by western blotting. Left panels represent the input and right panels represent bound proteins. Data are representative of three independent experiments.

Ubiquitylation involves recognition and binding of the E3 ligases to their protein targets (Pickart, 2001). The above results suggest that expression of NEDD4 and MARCH8 is required for efficient ubiquitylation of BST2. We thus determined whether this process involves binding of BST2 to these E3 ligases (Fig. 3A). HEK293T cells were transfected with an expression vector for FLAG–BST2 along with vectors encoding the hemagglutinin (HA)-tagged E3 ligases. Interaction of FLAG–BST2 with the HA-tagged E3 ligases was assessed by co-immunoprecipitation using an antibody against FLAG, followed by western blot analysis. No interaction was observed between FLAG–BST2 and HA–WWP1, used as a negative control (lower right panels), nor with β-TrCP (lower left panels), which is consistent with the lack of requirement of β-TrCP for BST2 ubiquitylation (Fig. 2A). On the contrary, FLAG–BST2 co-immunoprecipitated with HA–NEDD4, albeit weakly (upper left panels), as well as HA–MARCH8 (upper right panels). Epitope tagging of BST2 has been reported to alter some of its functions (Lv et al., 2014). Consequently, binding of these E3 ligases to exogenously expressed untagged BST2 in HEK293T cells (Fig. S1A), or endogenous BST2 in HeLa cells (Fig. S1B) was evaluated by co-immunoprecipitation using an antibody against BST2. Similarly, binding of BST2 with HA–MARCH8 was detected, as well as a weak but specific interaction with HA–NEDD4 (Fig. S1A,B), further confirmed for endogenous NEDD4 (Fig. S1C,D). Altogether, our results suggest that NEDD4 and MARCH8 could promote BST2 ubiquitylation via binding to BST2.

We wondered whether the two E3 ligases cooperate with each other in binding to and ubiquitylation of BST2. We thus tested the impact that depleting each ligase had on the binding of BST2 to the other enzyme in HeLa cells (Fig. 3B,C) and HEK293T cells (not shown). Binding of FLAG–BST2 to HA–NEDD4 or to HA–MARCH8 in cells in which MARCH8 and NEDD4 had been knocked down, respectively, was assessed by co-immunoprecipitation. Depletion of NEDD4 did not significantly impact on binding of FLAG–BST2 to HA–MARCH8, suggesting that NEDD4 expression is not required for BST2 interaction with MARCH8 (HA–MARCH8 panel, lanes 23 vs 17). Reciprocally, MARCH8 knockdown did not impair binding of FLAG–BST2 to HA–NEDD4 (Fig. 3C, HA–NEDD4 panel, lanes 22 vs 16). Taken together, it can be concluded that binding of BST2 by either NEDD4 or MARCH8 does not rely on the expression of the other ligase.

### NEDD4 and MARCH8 regulate BST2 turnover

**Fig. 4.**
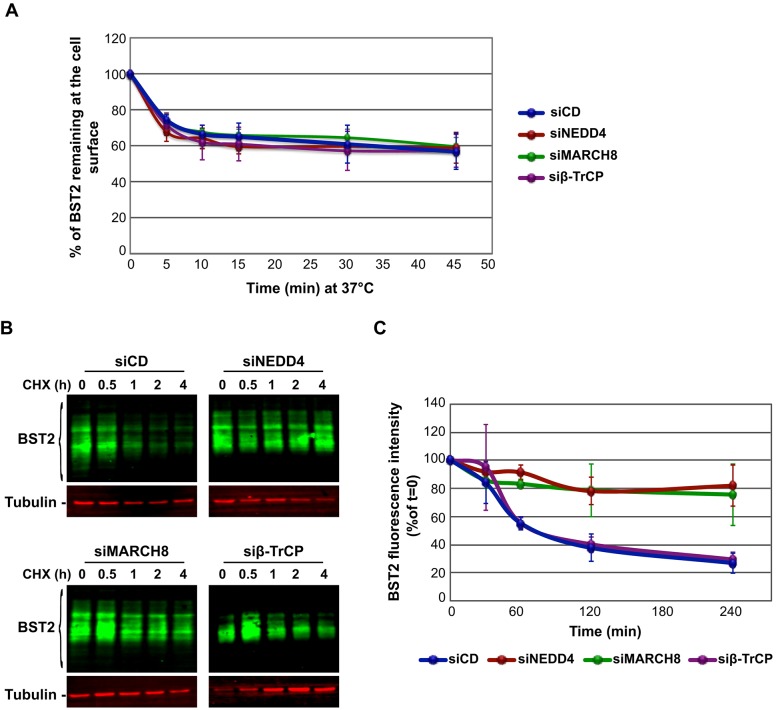
**Role of NEDD4 and MARCH8 in BST2 trafficking.** (A) NEDD4, MARCH8 and β-TrCP are not involved in the regulation of BST2 internalization. Internalization of BST2 in siRNA-transfected HeLa cells was monitored by immunofluorescence staining and flow cytometry analyses. The kinetics of BST2 internalization were determined as the amount of BST2 remaining at the cell surface at each time point compared to the amount of BST2 present at t=0 (set as 100%). Error bars represent the mean±s.d. (*n*=3). (B) NEDD4 and MARCH8 sort BST2 for degradation. siRNA-treated HeLa cells were incubated with cycloheximide for the times indicated above each lane. Equivalent amounts of each sample (20 µg of protein) were analyzed by quantitative western blotting using antibodies against BST2 and tubulin as a loading control. For each sample, the relative amount of BST2 was measured using ImageJ software and normalized to tubulin levels. Values at t=0 were set to 100% in the graph shown on the right. Error bars represent the mean±s.d. (*n*=3).

Ubiquitylation is involved in the regulation of many trafficking processes, such as internalization and sorting for degradation (Pickart, 2001). BST2 traffics between the TGN, the plasma membrane and endosomes, with a fraction targeted to lysosomes for degradation (Habermann et al., 2010; Masuyama et al., 2009; Rollason et al., 2007). NEDD4 has been shown to regulate the internalization, as well as endosomal sorting for degradation, of proteins such as connexin 43 and PTEN (Leykauf et al., 2006; Wang et al., 2007). MARCH8 has also been proposed to regulate internalization and sorting for degradation of cargos such as transferrin receptor (TfR), HLA-B7-2 and CD98 (Ablack et al., 2015; Bartee et al., 2004; Fujita et al., 2013; Ohmura-Hoshino et al., 2006a). We investigated, therefore, the role of the two E3 ligases, and by extension ubiquitylation, in the regulation of BST2 trafficking (Fig. 4). We first analyzed the consequences of depleting their expression on the kinetics of BST2 internalization (Fig. 4A). In control cells (siCD), almost 40% of BST2 present at the cell surface was internalized within 10 min, followed by a stabilization of the level of the pool of surface-labeled BST2, reflecting the balance between its internalization and recycling. In NEDD4- and MARCH8-depleted cells, the internalization rate of BST2 remained unchanged compared to control cells or to cells depleted for β-TrCP. This suggests that ubiquitylation of BST2 by the two E3 ligases is not involved in regulation of BST2 endocytosis.

We then assessed the role of the E3 ligases in BST2 sorting for degradation (Fig. 4B,C). The turnover of BST2 was monitored in siRNA-transfected HeLa cells after incubating the cells in growth medium containing cycloheximide, followed by quantitative western blot analysis of BST2 expression. Consistent with our previous studies (Janvier et al., 2011), almost 80% of BST2 was degraded within 4 h in cells that had been transfected with control siRNA (siCD). By contrast, in NEDD4- and MARCH8-depleted cells, the half-life of BST2 was prolonged, with only 20% of the initial BST2 pool degraded after 4 h (siNEDD4; siMARCH8). This stabilization might account for the increased amount of BST2 at steady state (Fig. 1C,D) and suggests that ubiquitylation of BST2 by NEDD4 and/or MARCH8 regulates its sorting for lysosomal degradation. Surprisingly, in cells depleted for β-TrCP, no major alteration of BST2 turnover was observed compared to control cells, leaving unsolved the mechanism responsible for the enhanced level of BST2 in these cells (Fig. 1C).

### Interplay between NEDD4 and MARCH8 in regulating BST2 trafficking

**Fig. 5.**
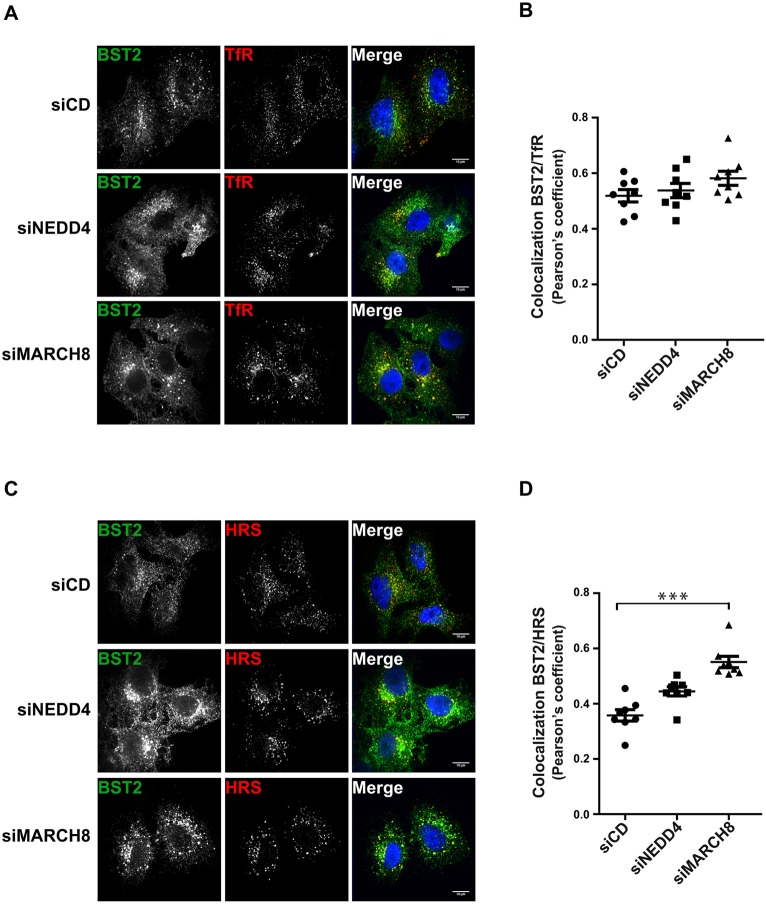
**Effects of NEDD4 or MARCH8 silencing on BST2 subcellular localization.** siRNA-transfected HeLa cells were permeabilized before fixation and immunostained with antibodies against BST2 (green) and transferrin receptor (TfR, early/recycling endosomes; red) (A) or HRS (endosomes/MVBs; red) (C) along with DAPI (blue) to visualize the nucleus of the cells. Cells were observed with a confocal microscope. Scale bars: 10 µm. (B,D) Colocalization between BST2 and TfR or BST2 and HRS was assessed by calculating the Pearson's correlation coefficient on eight images per condition. Each dot represents the Pearson's correlation coefficient of one image featuring at least three cells. Error bars represent the mean±s.e.m. from each image (*n*=3), ***P<0.001 (Student's *t*-test).

We have identified two E3 ligases, NEDD4 and MARCH8, that participate in BST2 ubiquitylation and subsequent sorting for degradation. We next assessed, by performing immunofluorescence (IF) analyses, the consequences of their depletion on the intracellular distribution of BST2 along with well-characterized markers of intracellular compartments (TfR – early/recycling endosomes; HRS – endosomes/MVBs; TGN46 – TGN; and LAMP1 – lysosomes) in HeLa cells (Fig. 5; Fig. S2). In cells depleted for NEDD4 or MARCH8, BST2 staining appeared to be more intense compared to that in control cells (siCD) (Fig. 5A,C; Fig. S2A,C), consistent with the enhanced amount of BST2 observed by western blotting (Fig. 1). In agreement with previous studies, in control cells, BST2 was present in TfR-, HRS- and TGN46-positive compartments [Pearson's correlation coefficient (Bolte and Cordelières, 2006): 0.52 for TfR, 0.36 for HRS and 0.39 for TGN46 (Fig. 5B,D and Fig. S2B)]. No striking colocalization of BST2 with LAMP1 was detected (0.27 for LAMP1; Fig. S2C,D), consistent with BST2 not being stably present in lysosomes. Analysis of NEDD4-depleted cells showed no profound alteration of BST2 distribution despite enhanced BST2 levels, with a coefficient of colocalization of BST2 with TfR, HRS and TGN46 similar to that of control cells (Fig. 5; Fig. S2). In contrast, cells that had been depleted of MARCH8 displayed a significant increase of the proportion of BST2 in clumped structures that were co-stained with HRS, suggesting an accumulation of BST2 in this compartment (Pearson's coefficient=0.55; Fig. 5C,D).

**Fig. 6.**
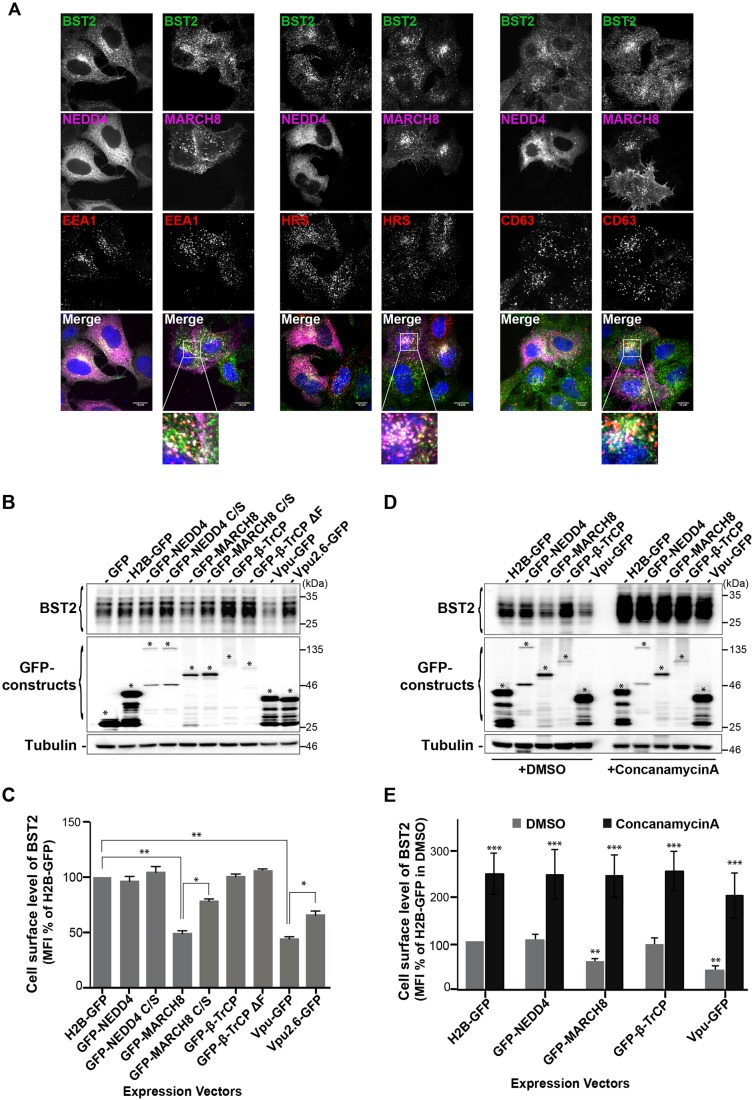
**BST2 distribution in cells overexpressing NEDD4 or MARCH8.** (A) HeLa cells that had been transfected with plasmids encoding GFP–NEDD4 or GFP–MARCH8 were permeabilized and co-stained with antibodies against BST2 (green) and EEA1, HRS or CD63 (red), along with DAPI (blue). Cells were then analyzed by confocal microscopy. Scale bars: 10 µm. Areas indicated in the square are magnified in the lower panels. (B) HeLa cells that had been transfected with the indicated expression vectors were lysed, and BST2 levels were assessed by western blot analyses. Tubulin is the loading control. Asterisks indicate the bands corresponding to GFP fusion proteins. These data are representative of three independent experiments. (C) HeLa cells that had been transfected with the indicated GFP fusion constructs were surface-stained with an antibody against BST2 or IgG1 isotype control and processed for flow cytometry analysis. The level of cell surface expression of BST2 was calculated as the mean fluorescence intensity (MFI) values obtained for BST2 staining minus MFI values of the isotype control. Values for each condition were normalized to those of control H2B–GFP expressing cells set as 100%. Bars represent the mean±s.d. (*n*=4); **P<0.01, *P<0.05 (Student's *t*-test). (D,E) HeLa cells expressing the indicated GFP fusion constructs for 12 h were further cultured for 16 h in the presence of either DMSO or 50 nM of Concanamycin A, an inhibitor of endosomal/lysosomal acidification, and processed for western blot and flow cytometry analyses as described in B,C. Bars represent the mean±s.d. (*n*=3); ***P<0.001, **P<0.01, *P<0.05 (Student's *t*-test).

We further analyzed the distribution of BST2 with the aforementioned intracellular markers in cells overexpressing GFP-tagged versions of NEDD4 or MARCH8 (Fig. 6). GFP–NEDD4 displayed a diffuse localization in cells, which prevented us from drawing any conclusion on its colocalization with BST2 (Fig. 6A). Furthermore, no potent alteration in the distribution and expression level of BST2 was observed in GFP–NEDD4-expressing cells (Fig. 6A-C). By contrast, and consistent with previous studies (Bartee et al., 2004; Goto et al., 2003), membrane-spanning MARCH8 was present in perinuclear endosomal compartments that were co-stained with HRS and CD63, with a fraction of MARCH8 present at the cell surface (Fig. S3). Analysis of the BST2 distribution showed an intense colocalization with MARCH8, mainly in HRS- and CD63-positive compartments (Fig. 6A), suggesting increased targeting of BST2 to MVBs upon MARCH8 overexpression. We also noticed in some cells that overexpression of MARCH8 induced decreased expression of BST2, which was further confirmed by western blot and flow cytometry analyses of overall and cell surface levels of BST2, respectively (Fig. 6B,C). In agreement with a previous report (Bartee et al., 2006), this indicates enhanced turnover of BST2 under these conditions. We next examined whether MARCH8-induced degradation of BST2 occurs in the lysosomes. HeLa cells overexpressing the E3 ligases were treated with DMSO or Concanamycin A (CMA), a vacuolar H(+)-ATPase inhibitor that blocks endosomal acidification and thus lysosomal degradation, and then analyzed the effects by western blotting and flow cytometry analyses (Fig. 6D,E). Compared to DMSO-treated cells, MARCH8 overexpression in cells treated with CMA no longer induced downregulation of BST2 level, which was similar to that observed in cells expressing H2B-GFP or GFP-NEDD4 or -β-TrCP, suggesting that MARCH8-induced downregulation of BST2 is mediated by lysosomal degradation.

### NEDD4 and MARCH8 are dispensable for Vpu-induced downregulation of BST2

**Fig. 7.**
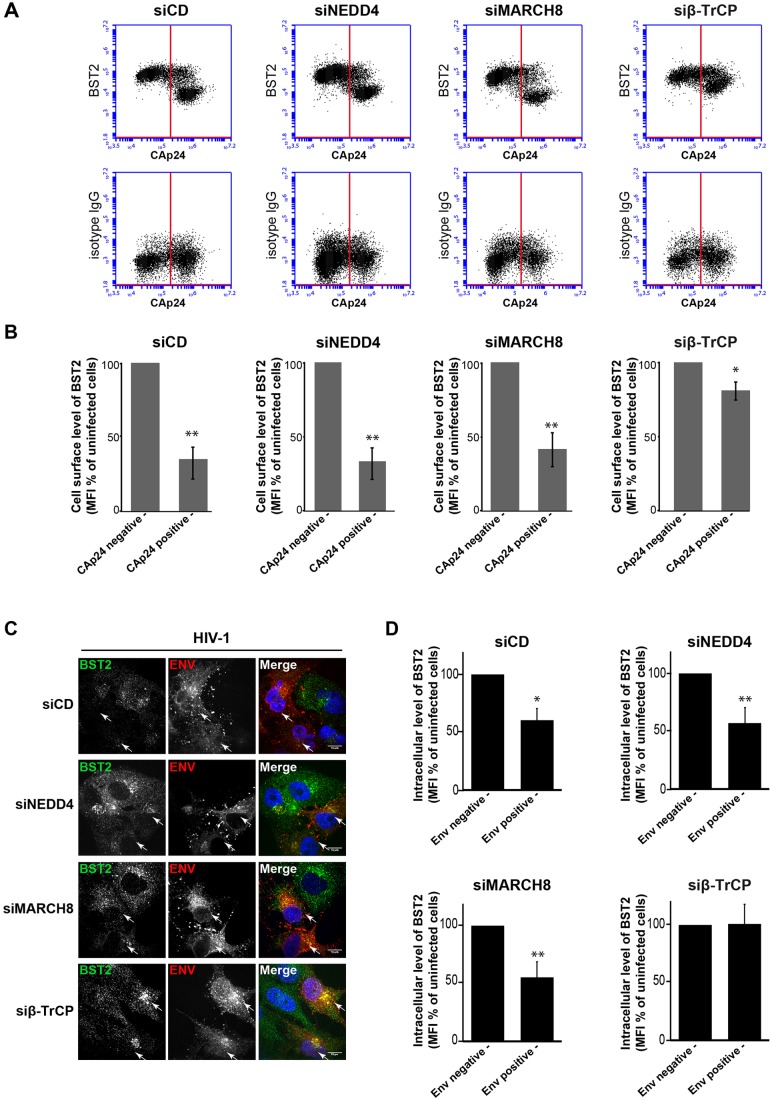
**NEDD4 and MARCH8 are not required for Vpu-induced BST2 downregulation.** (A,B) Effects of NEDD4, MARCH8 or β-TrCP silencing on Vpu-induced cell surface downregulation of BST2. HeLa cells that had been transfected with the indicated siRNA were infected with VSV-G-pseudotyped HIV-1 NL4-3 WT. Twenty-four hours later, cells were surface-stained for BST2 or an isotype IgG control. The cells were then fixed, permeabilized and stained for Gag using a FITC-conjugated monoclonal antibody against CAp24. The cells were then processed for flow cytometry analysis. (A) Dot plot. Vertical lines indicate the gates set using non-infected cells stained as indicated. Left gate, non-infected cells; right gate, infected cells. (B) Bar graph representation of the cell surface level of BST2 in CAp24-negative cells (left bars) and CAp24-positive cells (right bars) for each siRNA condition. Values are expressed as the mean fluorescence intensity (MFI) for BST2 staining minus those of the isotype control, normalized to those of non-infected cells set as 100%. Bars represent the mean±s.d. (*n*=3); **P<0.01, *P<0.05 (Student's *t*-test). (C,D) β-TrCP is required for Vpu-induced BST2 degradation. (C) Infected siRNA-treated HeLa cells were permeabilized before fixation, and intracellular BST2 (green) and HIV Env (red) were labeled with specific antibodies. Nuclei were stained with DAPI. Cells were imaged by confocal microscopy. Env staining discriminates infected cells (arrows) and non-infected cells. Scale bars: 10 µm. Images are representative of three independent experiments. (D) Infected siRNA-treated cells were fixed, permeabilized and stained for intracellular BST2 and HIV-1 Env using specific antibodies before analysis by flow cytometry. BST2 expression levels on infected and non-infected cells, respectively, were expressed as in B. Bars represent the mean±s.d. (*n*=3);**P<0.01, *P<0.05 (Student's *t*-test).

Vpu has been shown previously to increase BST2 ubiquitylation through recruitment of β-TrCP (Gustin et al., 2012; Tokarev et al., 2010) and target BST2 for ESCRT-mediated lysosomal degradation (Agromayor et al., 2012; Janvier et al., 2011). We analyzed, therefore, the involvement of NEDD4 and MARCH8 in Vpu-induced downregulation of BST2 (Fig. 7; Fig. S4).

We first assessed the effects of depleting each ligase on Vpu-induced cell surface downregulation of BST2 (Fig. 7A,B). Considering the role of β-TrCP in Vpu-induced downregulation of BST2, siRNA targeting β-TrCP was used as a control. siRNA-treated Hela cells were infected with HIV-1 NL4-3 WT (HIV-1 WT) or a virus defective for Vpu (HIV-1 Udel) pseudotyped with vesicular stomatitis virus glycoprotein (VSV-G) that enables virus entry through endocytosis. Cell surface levels of BST2 were then assessed by flow cytometry analysis (Fig. 7A,B). Intracellular staining of the HIV-1 Capsid protein (CAp24) was used to distinguish infected (Fig. 7A,B, right gates and bars) from non-infected cells (left gates and bars). Depletion of NEDD4 or MARCH8 did not significantly alter the ability of Vpu to downregulate BST2 at the cell surface. Indeed, Vpu expression induced a ∼twofold decrease of BST2 cell surface expression, as observed in HIV-1 WT-infected control cells (siCD). On the contrary and consistent with previous studies (Douglas et al., 2009; Mitchell et al., 2009), depletion of β-TrCP significantly altered the ability of Vpu to downregulate BST2 from the cell surface compared to control cells, confirming that β-TrCP is required for this activity of Vpu.

We then analyzed the ability of Vpu to target BST2 for degradation following depletion of NEDD4, MARCH8, or β-TrCP (Fig. 7C,D). Because cells that had been depleted for NEDD4 or MARCH8 displayed low permissiveness to infection by HIV-1 (Fig. S4), these analyses were performed using IF (Fig. 7C) and flow-cytometry (Fig. 7D) analyses to allow identification of infected cells. siRNA-treated infected cells were permeabilized and stained for BST2 and the HIV-1 envelope protein (Env) to distinguish infected cells. Microscopy and flow cytometry analyses of control cells (siCD) showed a decreased intensity of BST2 staining when infected with HIV-1, compared to in the neighboring uninfected cells. A similar decrease in BST2 staining intensity was observed in HIV-1-infected cells that had been depleted for both NEDD4 and MARCH8, suggesting that the decrease in BST2 is due to Vpu recruitment of β-TrCP (Douglas et al., 2009; Mangeat et al., 2009; Mitchell et al., 2009). Consistently, cells that had been depleted of β-TrCP and infected with HIV-1 did not show degradation of BST2. Indeed, similar levels of BST2 were observed in infected cells compared to in uninfected neighboring cells (Fig. 7C,D, lower panels). These results were further confirmed by western blot analysis (Fig. S4) that also showed a noticeable modification of the BST2 profile in β-TrCP-depleted cells infected with WT virus compared to in cells infected by HIV-1 Udel virus (Fig. S4, lane 5 vs 10) or in control cells (siCD, lane 2). This indicates that Vpu uses β-TrCP to induce BST2 downregulation, and bypasses NEDD4 and MARCH8, which are involved in constitutive turnover of BST2.

### Vpu connects BST2 to β-TrCP without interfering with BST2 binding to NEDD4 or MARCH8

**Fig. 8.**
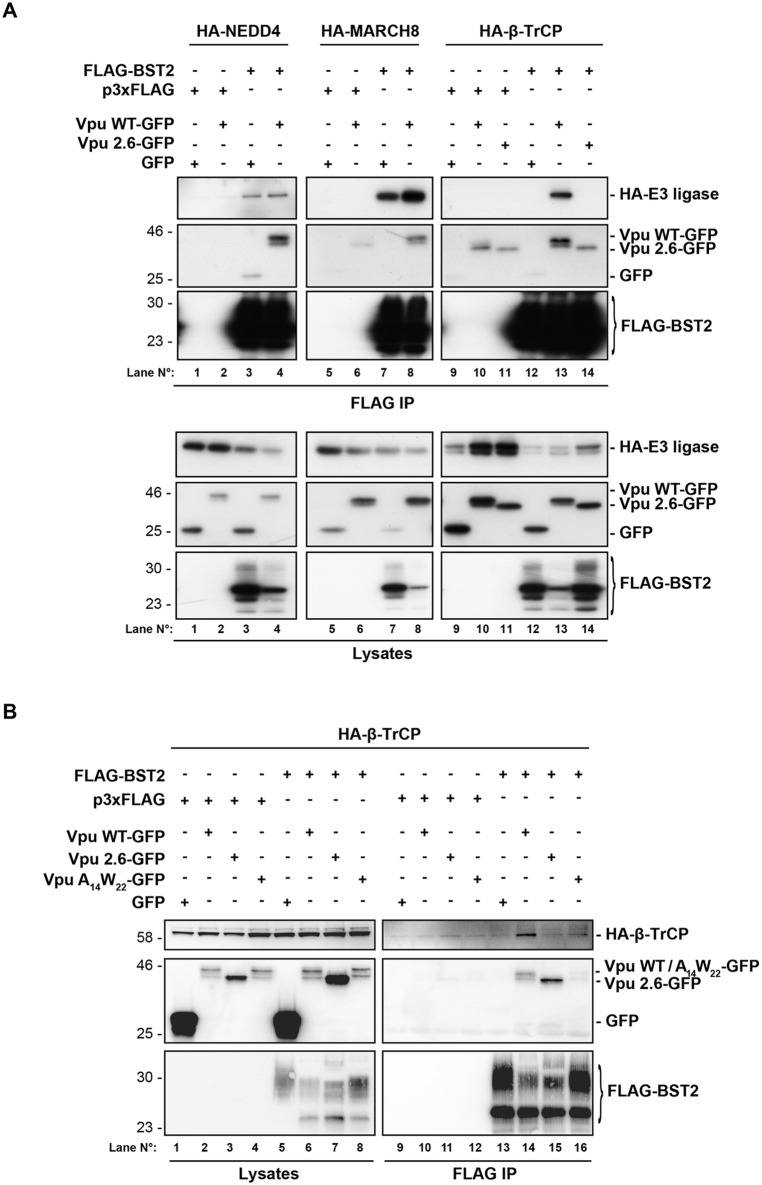
**Vpu connects BST2 to β-TrCP.** (A) HEK293T cells were transfected with plasmid encoding FLAG–BST2 or p3xFLAG vector (control) along with expression vectors for HA–NEDD4, HA–MARCH8 or HA–βTrCP, and either Vpu-WT–GFP or Vpu2.6–GFP, which cannot bind to β-TrCP, or GFP (as control). Binding of FLAG–BST2 with HA-tagged E3 ubiquitin ligases and Vpu–GFP proteins was assessed by immunoprecipitation (IP) of FLAG–BST2 followed by western blot analyses. Upper panels represent bound proteins and lower panels represent the input. Data are representative of four independent experiments. (B) Interaction between FLAG–BST2 and HA–β-TrCP in the presence of Vpu-WT–GFP, Vpu2.6–GFP or mutated Vpu A14L-W22A (Vpu A_14_W_22_–GFP), which is impaired in the ability to bind to BST2, was assessed as described in A. Left panels represent the input and right panels represent bound proteins. Data are representative of two independent experiments.

To further decipher the molecular mechanism involved, we investigated the impact of Vpu expression on the interaction of BST2 with its E3 ligases by co-immunoprecipitation, as one might speculate that Vpu interferes with the binding of BST2 to its E3 ligases in favor of β-TrCP. No major alteration in FLAG–BST2 binding to HA–NEDD4 (Fig. 8, lane 4) or HA–MARCH8 (lane 8) was observed upon expression of Vpu–GFP, compared to GFP-expressing control cells (lanes 3 and 7, respectively). This indicates that the viral protein does not interfere with the binding of BST2 to the two E3 ligases involved in its sorting for degradation. In cells expressing Vpu–GFP, we did detect binding of FLAG–BST2 to HA–β-TrCP (lane 13), which was not observed in cells expressing GFP alone (lane 12) nor in cells expressing Vpu mutated at residues S52 and S56 (Vpu2.6–GFP), which is unable to bind to β-TrCP (lane 14) (Margottin et al., 1998), suggesting that Vpu connects BST2 to β-TrCP (Mangeat et al., 2009). To confirm this model, co-immunoprecipitation between FLAG–BST2 and HA–β-TrCP was assessed in cells expressing Vpu mutated on the residues W22 and A14, which are required for its interaction with BST2 (Skasko et al., 2012; Vigan and Neil, 2010) (Fig. 8B). As expected, interaction of FLAG–BST2 with HA–β-TrCP was affected in cells expressing the double mutant W22A-A14L (VpuW_22_A_14_–GFP) (lane 16), compared to in cells expressing WT Vpu (lane 14), consistent with the notion that Vpu bridges BST2 to β-TrCP.

Altogether, our data suggest that Vpu connects the restriction factor BST2 to β-TrCP, inducing its downregulation, without interfering with the binding of BST2 to the E3 ligases NEDD4 and MARCH8, which are involved in its constitutive trafficking and turnover.

## DISCUSSION

Ubiquitylation regulates virtually every cellular process and therefore cellular homeostasis and fitness (Pickart, 2001). The ubiquitylation pathway is commonly manipulated by pathogens to propagate and to evade the host cell immune defense (Randow and Lehner, 2009). In this study, we have highlighted the role of two E3 ligases, NEDD4 and MARCH8 in the regulation of BST2 ubiquitylation, trafficking and delivery to the lysosomes for degradation. We showed that NEDD4 and MARCH8 overexpression increases BST2 ubiquitylation, an effect that is abrogated upon mutation of their catalytic sites (Fig. 2C,D). Conversely, NEDD4 and MARCH8 depletion decreased BST2 ubiquitylation (Fig. 2A,B) and resulted in its delayed turnover (Fig. 4B,C). Surprisingly, both E3 ligases appeared to be dispensable for the ability of Vpu to promote cell-surface downregulation and degradation of BST2 (Fig. 7). This activity requires the recruitment of β-TrCP, which does not potently contribute to the regulation of basal ubiquitylation and turnover of BST2 (Figs 2, 4 and 7), suggesting that the viral protein does not hijack the cellular machinery involved in constitutive ubiquitylation and turnover of BST2 but usurps an alternative machinery for this process.

Our results unravel the role of NEDD4 and MARCH8 in the regulation of constitutive ubiquitylation and trafficking of BST2 within the endocytic pathway (Figs 2, 4 and 5). Depletion of each E3 ligase independently altered BST2 sorting for degradation (Figs 2A,B and 4B), with no alteration in the internalization rate of BST2 from the PM (Fig. 4A). This suggests that the alteration in BST2 trafficking most likely occurs at the endosome level, and by extension, that NEDD4 and MARCH8 regulate endosomal sorting of BST2. Knockdown of these E3 ligases resulted in an overall increase in BST2 levels (Fig. 1C,D). However, NEDD4-depleted cells displayed no major alteration of BST2 intracellular distribution (Fig. 5). Depletion of MARCH8, by contrast, induced an enhanced distribution of BST2 on an HRS-positive compartment, suggesting retention of the protein in this compartment (Fig. 5). Consequently, one might propose that MARCH8 acts downstream of NEDD4 on BST2 ubiquitylation and trafficking along the endosomal pathway. The delayed turnover of BST2 observed upon depletion of NEDD4 might result from enhanced and sustained recycling of BST2 from early endosomes to the cell surface. Later in the endosomal pathway, MARCH8 present at the level of the MVB (Fig. S3) might interact with BST2 and trigger its ubiquitylation and sorting from this compartment. This notion is supported by (1) the build-up of BST2 in an HRS-positive compartment upon depletion of MARCH8 (Fig. 5), suggesting that BST2 is unable to recycle back to the plasma membrane, and (2) its redistribution into HRS-positive endosomes upon overexpression of MARCH8 leading, ultimately, to reduced levels of BST2 (Fig. 6).

We also observed an increased level of BST2 in cells that had been depleted of β-TrCP, without alteration of BST2 turnover and ubiquitylation (Figs 1, 2 and 4). β-TrCP regulates proteasome-associated quality control of proteins in the biosynthetic pathway, as well as endo/lysosomal degradation of proteins (Fuchs et al., 2004). One might propose that depletion of β-TrCP induces a ubiquitin-dependent alteration of a yet-to-be-determined regulator of BST2 in the ER and/or at the endo/lysosomal pathway at a cryptic level, leading ultimately to an increase of BST2 levels during the course of our experiments.

Substrate recognition by NEDD4 relies on the interaction of its WW domain with a proline rich PPxY (PY) motif within its substrates (Boase and Kumar, 2015). BST2 does not harbor a typical PY motif, suggesting the potential involvement of an adaptor protein for its interaction with NEDD4. Examples of adaptors for NEDD4 binding have been described, such as Ndfip (Boase and Kumar, 2015), or members of the Arrestin family (Becuwe et al., 2012). WW domains can also interact with phosphorylated serine/threonine residues in substrates (Lu et al., 1999; Sudol et al., 1995), and it is noteworthy that BST2 contains two serine residues and one threonine (S3-T4-S5) in its cytoplasmic tail. Moreover, albeit controversial (Gustin et al., 2012), these residues have been proposed to be the targets of Vpu-induced ubiquitylation of BST2 (Tokarev et al., 2010). Their contribution to BST2 binding to NEDD4, therefore, would be worth testing. To date, no specific determinants for MARCH8 recognition have been characterized. Of note, interaction of Myc–BST2 with MARCH8–GFP has been previously addressed by pulling down MARCH8, but this did not reveal any interaction between the two proteins (Fujita et al., 2013). This discrepancy suggests that BST2 might not be one of the main partners of MARCH8 and/or, alternatively, that the interaction between both proteins is very transient.

MARCH8 (initially called c-MIR) was first identified as the cellular functional homolog of the KSHV membrane-bound ubiquitin ligases K3 and K5 (also called MIR1 and MIR2 for ‘modulator of immune response’) (Goto et al., 2003). Antagonism of BST2 antiviral activity by the K5 ligase relies on ubiquitylation of BST2 on lysine residue 18 located in its cytoplasmic tail and subsequent sorting of the restriction factor for ESCRT-dependent lysosomal degradation (Bartee et al., 2006; Pardieu et al., 2010). We have previously shown that BST2 undergoes ESCRT-mediated targeting to the lysosome (Janvier et al., 2011) and have demonstrated herein the role of MARCH8 in BST2 ubiquitylation and delivery to lysosomal degradation. Altogether, this supports the view that the K5 ligase mimics the endogenous cellular activity of MARCH8 to counteract BST2 restriction activity towards KSHV infection. Further characterization of the role of the lysine residue of BST2 in mediating its sensitivity to MARCH8-induced ubiquitylation will be required to confirm the mechanistic resemblance between both cellular and viral proteins towards BST2 regulation.

Vpu favors ESCRT-mediated targeting of BST2 to the lysosomes (Agromayor et al., 2012; Janvier et al., 2011). This process has been shown to be ubiquitin dependent (Douglas et al., 2009; Goffinet et al., 2010; Mitchell et al., 2009; Tokarev et al., 2010), but does not involve hijacking of the machinery involved in the regulation of constitutive ubiquitylation and sorting of BST2 for degradation (Fig. 7). Indeed, depletion of NEDD4 or MARCH8 had no impact on the ability of Vpu to modulate the expression of BST2, an activity that relies on the recruitment of β-TrCP by Vpu (Figs 7 and 8), as previously reported (Mangeat et al., 2009; Mitchell et al., 2009; Tokarev et al., 2010). Vpu has been proposed to promote polyubiquitylation of BST2 on its serine and threonine residues (Tokarev et al., 2010). Our data suggest that NEDD4 and MARCH8 promote mono- and multi-ubiquitylation of BST2. This might explain the irrelevance of both E3 ligases on Vpu-induced sorting of BST2 for degradation.

Vpu-induced downregulation of BST2 has been suggested to be dispensable in counteracting BST2 antiviral activity, which mainly requires active removal of BST2 from the HIV-1 budding sites by the viral protein (Chu et al., 2012; Goffinet et al., 2010; Lewinski et al., 2015). The requirement of β-TrCP and consequent ubiquitylation of BST2 in Vpu-induced antagonism of BST2 antiviral activity remains controversial (Iwabu et al., 2009; Kueck et al., 2015; Mangeat et al., 2009; Mitchell et al., 2009). We did not observe any potent effect of β-TrCP depletion on the amount of HIV-1 released into cell supernatants (Fig. S4). However, these cells displayed increased expression of viral components, notably Env and Vpu, suggesting either increased sensitivity to HIV infection or enhanced transcription/translation of the viral genome that precludes drawing any conclusion about the involvement of β-TrCP on Vpu-mediated viral release. Along the same line of thought, measurement of virus production in cells that had been treated with siRNA against NEDD4 or MARCH8 did not allow us to draw any conclusions due to the low permissiveness of these cells to HIV-1 infection (Fig. S4). Interestingly, a recent paper has revealed the importance of MARCH8 in the regulation of HIV-1 Env glycoprotein trafficking and incorporation into budding viruses (Tada et al., 2015). Countering of BST2 antiviral activity by most strains of HIV-2 and a subset of SIV strains (SIV_agm_Tan and SIV_mac239_Δ*nef* isolates) is performed by Env glycoproteins (Gupta et al., 2009; Jia et al., 2009; Le Tortorec and Neil, 2009; Serra-Moreno et al., 2011). Future work will explore in-depth the role of MARCH8 in HIV-2 Env-induced antagonism of BST2.

In summary, this study has highlighted two additional regulators of BST2, namely NEDD4 and MARCH8, which provides greater understanding of the mechanisms underlying BST2 turnover in cells under basal conditions Furthermore, our data show that Vpu bypasses the machinery that is constitutively involved in BST2 ubiquitylation and sorting for degradation; instead, Vpu favors recognition of the restriction factor by recruiting β-TrCP to trigger lysosomal targeting of BST2. Future studies will decipher the molecular and cellular mechanisms underlying regulation of BST2 expression and trafficking by Vpu.

## MATERIALS AND METHODS

### Cell culture

HeLa (National Institutes of Health; AIDS Reagent Program) and HEK293T (American Type Culture Collection) cells were grown in Dulbecco's modified Eagle's medium plus glutamine, antibiotics and 10% decomplemented fetal bovine serum (FBS) (Gibco^®^, Life Technologies).

### Recombinant DNA and transfection

The cDNAs for NEDD4 WT, catalytically inactive NEDD4 C867S mutant (gifts from Dr Peter Snyder, University of Iowa, USA), MARCH8 WT and catalytically inactive MARCH8 C/S (in which cysteine residues 83, 86, 123 and 126 were mutated to S) (gifts from Drs Adrian P. Kelly and Martin Jahnke, University of Cambridge, UK) were cloned into pEGFP-C2 vector (Clontech, France). Expression vectors for BST2, WT and mutated NEDD4 and MARCH8 fused to the HA or the FLAG affinity tags were obtained by cloning the cDNAs into pAS1B vector (Selig et al., 1999) or p3xFLAG vector (Janvier et al., 2011), respectively, enabling N-terminal tagging of the proteins. Expression vectors for GFP- or HA-tagged β-TrCP WT and the F-box deletion mutant (β-TrCPΔF) were obtained from Dr Florence Margottin-Goguet (Margottin et al., 1998). The NL4-3 Vpu mutants S52N-S56N (Vpu2.6) and A14L-W22A were made by performing PCR mutagenesis using the QuikChange II site-directed mutagenesis kit (Stratagene). WT and mutant cDNAs of NL4-3 Vpu were cloned into pEGFP-N1 vector (Clontech). Transfection of expression vectors into HeLa and HEK293T cells was performed using Lipofectamine LTX (Life Technologies) following the manufacturer's instructions.

### siRNA transfection

Cells were transfected with 5–30 nM siRNA using Lipofectamine RNAiMAX (Life Technologies) according to the manufacturer's instructions and analyzed 4 days post transfection. The 21-nucleotide RNA duplex targeting both β-TrCP1 and β-TrCP2 (5′-GUGGAAUUUGUGGAACAUCdTdT-3′ at positions 448–465 and 265–285, respectively) was designed as previously described (Jin et al., 2003; Guardavaccaro et al., 2003) and synthesized by Dharmacon. The On-target plus SMART-pool siRNAs targeting BST2 (# L-011817-00), NEDD4 (#L-007178-00), NEDD4-L (#L-007187-00), MARCH8 (#L-007161-00), CBL (#L-003003-00), ITCH (#L-007196-00), WWP1 (#L-004251-00) were purchased from Dharmacon. The On-target plus non-targeting siRNA 1 (#D-001810-01 from Dharmacon) was used as control.

### SDS-PAGE and western blot analyses

SDS-PAGE and western blotting were performed as described previously (Caillet et al., 2011). Antibodies used are listed in Table S1.

Quantitative western blots for BST2 and tubulin were performed by incubating the membranes with rabbit anti-BST2 (National Institutes of Health) and mouse anti-tubulin (Sigma-Aldrich) antibodies, followed by incubation with Dylight™-800-conjugated anti-Rabbit IgG and Dylight™-680-conjugated anti-Mouse IgG (KPL-Thermo Scientific). The corresponding signals were then acquired using the Odyssey^®^ Infrared Imaging System (Li-Cor) for further quantification using ImageJ software.

### Ubiquitylation assay

HeLa cells transfected with siRNA or GFP-tagged constructs were lysed for 30 min at 4°C using lysis buffer [50 mM Tris-HCl, pH 7.6, 300 mM NaCl, 2 mM EDTA, 1% (v/v) NP-40, 0.1% (v/v) sodium deoxycholate and 0.2% SDS], supplemented with 20 mM *N*-ethylmaleimide (Calbiochem) and complete protease inhibitor Cocktail (Roche Diagnostics). Cell extracts were immunoprecipitated using BST2 antibody (2E6, Abnova) coupled to Dynabeads^®^ Protein G (Life Technologies). Immunoprecipitates were resolved on SDS-PAGE before immunoblotting using antibodies to ubiquitin (HRP-linked FK2 antibody, Enzo Life Sciences) and BST2 (National Institutes of Health).

### Cycloheximide treatment

BST2 turnover was assessed by incubating the cells with cycloheximide (Calbiochem), as previously described (Janvier et al., 2011), followed by quantitative western blot analyses using the Odyssey^®^ Infrared Imaging System (Li-Cor).

### Co-immunoprecipitation experiments

Cells were harvested 24 h after transfection and lysed for 30 min at 4°C in lysis buffer (50 mM Tris-HCl, pH 7.6, 2 mM EDTA, 150 mM NaCl, 0.5% NP-40) complemented with protease inhibitor cocktail. The cell lysates were immunoprecipitated with anti-FLAG M2 affinity Gel (Sigma-Aldrich) or anti-BST2 (2E6) antibody or the corresponding IgG isotype (Biolegend) coupled to Dynabeads^®^ Protein G for 4 h at 4°C. Purified proteins were resolved by SDS-PAGE followed by western blotting using anti-HA conjugated to horseradish peroxidase (HRP) (Roche^©^ Diagnostics), anti-FLAG^®^M2 conjugated to HRP (Sigma-Aldrich), anti-GFP conjugated to HRP (Genetex), anti-BST2 (National Institutes of Health) or anti-NEDD4 (Millipore) antibodies.

### Immunofluorescence microscopy

Immunofluorescence analyses in cells that had been transfected with siRNA or GFP-tagged constructs and/or infected were performed as described previously (Caillet et al., 2011). Antibodies used are listed in Table S2. Coverslips were mounted in Fluoromount-G (SouthernBiotech).

Cells were analyzed with a Leica DMI6000B spinning disk microscope. Series of 0.2 µm optical sections were recorded, and images were processed using ImageJ software. Quantitative colocalization analysis was performed with the JACoP tool (ImageJ software) using calculation of Pearson's correlation coefficient (Bolte and Cordelières, 2006). The Pearson's coefficient was calculated for eight images per condition, with about three to five cells per image.

### Flow cytometry

Cells that had been transfected with plasmids encoding GFP-tagged constructs were stained 24 h after transfection with Alexa-Fluor-647-conjugated anti-BST2 antibody (clone RS38E, Biolegend) or control IgG1 (Biolegend) in PBS with 1% BSA for 1 h at 4°C. Cells were washed and fixed in PBS with 1% BSA and 1% paraformaldehyde (PFA, Electron Microscopy Science) before analysis using the Cytomics FC500 Flow Cytometer (Beckman Coulter) or the Accuri™ C6 Flow Cytometer (BD Biosciences). Gates for GFP were set using non-transfected cells. All the data were analyzed using the CXP software or the C6 cytometer software.

Surface staining of BST2 in HIV-1-infected cells was performed as described above. Cells were then fixed, permeabilized and stained with a FITC-conjugated anti-CAp24 antibody (Beckman Coulter) before analysis.

Intracellular staining of infected cells was performed in permeabilized fixed cells using antibodies against BST2 (2E6, Abnova) and HIV-1 Env (2G12, National Institutes of Health), followed by staining with the appropriate secondary antibodies (Table S2) before analysis.

### Internalization assay

siRNA-transfected cells were surface-stained with anti-BST2 antibody (RS38E, Biolegend) or IgG1 isotype control for 1 h at 4°C in growth medium (DMEM+FBS 10%). Cells were washed three times in cold medium, an aliquot representing t=0 was kept at 4°C. The remaining cells were transferred to 37°C for different periods of time to allow internalization of BST2. At each time point, cells were transferred in cold medium then stained with Alexa-Fluor-647-conjugated anti-mouse secondary antibody for 30 min at 4°C, washed three times and fixed in PBS with 1% BSA and 1% PFA before analysis by flow cytometry. The BST2 internalization rate was calculated as the amount of BST2 present at the cell surface at each time point compared to the amount present at t=0.

### RNA extraction and RT-qPCR

RNA extractions and RT-qPCR analyses were performed as described previously (Madjo et al., 2016). RNAs that had been extracted using the QIAGEN RNeasy Mini kit and treated with DNAse (Qiagen, RNAse-free DNase set) were reverse transcribed using the High Capacity Reverse Transcription kit (Applied Biosystem) and quantified using real-time PCR using the Roche LightCycler 480 SYBR Green 1 Master kit (Roche Diagnostics) and specific primers. Each sample was analyzed in technical triplicates. The relative abundance of each mRNA tested was normalized to that of the GAPDH mRNA level. The primers used were: MARCH8 (5′-ACAGGAAGCCTCCACTTCG-3′, 5′-GAGTCACTGTAAGGTGCAG-3′), βTrCP1 (5′-CCAACATGGGCACATAAACTCG-3′, 5′-CCTACGGTTTAGTGATACACGACG-3′), βTrCP2 (5′-ACGAATGGTACGACGCACTGATGATCC-3′, 5′-CCTACACTTGTGCCCACTTCA-3′) and GAPDH (5′-GCATGGACTGTGGTCATGAG-3′, 5′-TGCACCACCAACTGCTTAGC-3′).

### Viral stocks and HIV-1 production assay

Stocks of HIV-1 NL4-3 WT (National Institutes of Health, AIDS Reagent Program) and HIV-1 NL4-3 Udel (from Dr. Klaus Strebel, National Institutes of Health, Bethesda, USA), pseudotyped with VSV G glycoprotein were prepared as previously described (Janvier et al., 2011).

HIV-1 production assays were performed as described (Janvier et al., 2011). Pelleted viruses and cell lysates were analyzed by western blotting using anti-CAp24 (ARP366), anti-SUgp120 (110H), anti-TMgp41 (41A), anti-Vpu, anti-BST2 and anti-tubulin antibodies (see Table S1).

### Statistical analysis

Statistical significance was analyzed using paired two-tailed Student's *t*-test and the one-way ANOVA test, and expressed as a *P*-value. Each experiment was performed independently at least three times.
